# Alcohol and Drug Use Prevalence and Factors Associated With the Experience of Alcohol Use in Iranian Adolescents

**DOI:** 10.5812/ircmj.4022

**Published:** 2013-03-05

**Authors:** Azam Baheiraei, Zeinab Hamzehgardeshi, Mohammad Reza Mohammadi, Saharnaz Nedjat, Eesa Mohammadi

**Affiliations:** 1 Department of Reproductive Health, Tehran University of Medical Sciences, Tehran, IR Iran; 2 Center for Community-Based Participatory Research, Tehran University of Medical Sciences, Tehran, IR Iran; 3 Department of Midwifery, Mazandaran University of Medical Sciences, Sari, IR Iran; 4 Department of psychiatric, Psychiatry and Psychology Research Centre, Tehran University of Medical Sciences, Tehran, IR Iran; 5 School of Public Health, Knowledge Utilization Research Center, Tehran University of Medical Sciences, Tehran, IR Iran; 6 Department of Nursing, Tarbiat Modares University, Tehran, IR Iran

**Keywords:** Alcohol Drinking, Prevalence, Adolescent, Iran

## Abstract

**Background:**

Alcohol and other drugs use is a problem among adolescents leading to numerous physical, social, and educational damages.

**Objective:**

For determining the prevalence of alcohol and other substance use as well as the factors associated with the experience of alcohol use in adolescents.

**Patients and Methods:**

This is a population-based and cross-sectional study, which was conducted in August 2010 on adolescents aged 15–18 years in Tehran. Data were collected by a Youth Risk Behavior Surveillance System (YRBSS) in 1,201 adolescents. The multistage cluster sampling method was used. Questions belonging to the domain of alcohol and other substance use were analyzed.

**Results:**

In general, 15.1% of adolescents had experienced alcohol, which is significantly higher in boys (21.9%) compared to girls (8.4%) (P = 0.000). 3.1% of adolescents had experience using opium and marijuana. 5.6% had used ecstasy. The results of multivariate logistic regression indicated that low parental control rather than medium control [AOR: 0.09], lifetime cigarette use [AOR: 10.41], having a tobacco user friend [AOR: 4.36], and having an alcohol user friend [AOR: 5.84] are factors that are significantly related to the experience of alcohol use in female adolescents. In addition, studying in private schools rather than public schools [AOR: 3.46], lifetime cigarette use [AOR: 3.41], lifetime water pipe use [AOR: 4.43], experience of sexual activity [AOR: 8.52], having an alcohol user friend [AOR: 12.60], and having a water pipe user in family [AOR: 2.98] are factors that are significantly related to the experience of alcohol use in male adolescents.

**Conclusions:**

We recommend interventional plans based gender aimed at improving adolescent health with regard substance abuse.

## 1. Background

Alcohol and other drugs use is a problem among adolescents,(1) leading to numerous physical, social, and educational damages. For example, substance abuse in adolescence is associated with adverse outcomes in youth, such as addiction, depression, suicide, interpersonal problems with family and peers, driving-related injuries or death, and detrimental impacts on the economy of the family and society (2, 3). Currently, substance abuse is growing among adolescents worldwide (4). In general, alcohol is the most common substance abused by European students, followed by hashish (5). In an Iranian study conducted on students in Shiraz, the use of alcohol and psychedelics was reportedly 32% and 2.2% respectively (6). Similarly, abusing substances such as alcohol, opium, ecstasy, and heroin was as frequent as 12.6%, 1.4%, 0.7%, and 0.3%, respectively, among students in Rasht, Iran (7). Moreover, 12.7% and 2% of the second-grade high school students in Tabriz, Iran, reported to have experienced alcohol and psychedelics (8). Previous studies indicate that over 90% of substance abusers start their experience in adolescence (9). Therefore, monitoring and controlling during adolescence is essential for reducing substance abuse in adulthood (10). Identifying the extent of the problem and its associated factors in adolescents is the first step towards understanding the related factors. Since cultural limitations have hindered a comprehensive understanding of the prevalence of alcohol and other drugs’ use among adolescents in Tehran.

## 2. Objective

We undertook the present study to assess the prevalence of using alcohol and other substances as well as the factors influencing the experience of alcohol use among adolescents aged 15–18 years.

## 3. Patients and Methods

This is a cross-sectional, population-based study aimed at determining the prevalence of alcohol and other substance use as well as the factors affecting the experience of alcohol use in adolescents. Our data collection tools consisted of questions in the domain of alcohol and other substances use of the Youth Risk Behavior Surveillance System (YRBSS) developed by the Centers for Disease Control and Prevention (11) as well as a socio-demographic questionnaire developed by the authors; both questionnaires were completed by the participants in a self-administered fashion. The reliability of the modified version of the questionnaire was evaluated by administering a test re-test to 98 adolescents aged 15–18 years, recruited through the convenience approach. In order to evaluate the reliability, we used the mean Intra class Correlation Coefficient (ICC) and Cronbach’s α. For the questions in the domain of alcohol and other drugs abuse, the mean ICC value was 0.76 (0.46–0.95), and the mean Cronbach’s α was 0.86, indicating an acceptable reliability for the questionnaire (12).The current study is a part of the large study that determined adolescent risk behaviors in Tehran, Iran. The estimated sample size of this study is 1200 adolescents aged 15-18 years living in the households in the 22 main municipal sectors of Tehran.

To determine the sample size in the quantitative phase, the following calculation was done:

N = Z^2^ 1-α/2×P (1- P) / d^2^

With regard to type 1 error (alpha) of 5% and accuracy of 0.5 and the most common high-risk behavior (tobacco use, especially water pipe experience) of previous researches that has been 30.6%, approximately 300 samples are calculated. Due to socioeconomic differences in different districts of Tehran, the city was divided into four parts (300 × 4), reached a sample size of 1200 adolescents. The sampling method was multistage random cluster sampling. It must be noted that we have provided ample reassurance to each adolescent that their information will remain confidential throughout the process of data collection, analysis, and publication. A total of 1,234 adolescents completed the questionnaires. In addition, 33 questionnaires were more than 20% unanswered questions. We analyzed data from 1,201 questionnaires by using descriptive and analytic indices on SPSS version 16 and STATA version 10. We used multivariate logistic regression for an adjusted analysis. We considered the experience of alcohol use as the dependent variable and age; type of school; wealth index; parental education; parental control on the adolescent; level of parental supervision on friend selection by the adolescent; parental use of punishment, history of consulting with a consultant, a teacher, or an adult due to behavioral problems; parental inclination towards boys; decision maker of the family; adolescent’s success in education; adolescent’s inclination towards education; family income sufficiency; social class according to paternal occupation; experience of sexual contact; experience of cigarette and water pipe use; experience of narcotics use; having alcohol-using friends, having narcotics-using friends; having tobacco-using friends; and having a family member who uses tobacco or a water pipe as independent variables. P values of less than 0.05 were considered significant. In addition, variables that obtained p values of less than 0.2 on univariate analysis were entered into the multivariate logistic regression analysis model. The Ethics Committee of Tehran University of Medical Sciences approved the protocol of the study.

## 4. Results

### 4.1. Characteristics of the Participants

The mean age of the 1201 participants was 16.74 (SD = 1.09). Among adolescent, 90.5% was student. 56.6% was studying in public schools. 68.4% was middle social class.

### 4.2. Prevalence of Alcohol and Other Substance Use

Table 1 presents the prevalence of alcohol and other substance use based gender. In general, 15.1% of adolescents had experienced alcohol, which is significantly higher in boys (21.9%) compared to girls (8.4%) (P = 0.000; odds ratio: 3.04; 95% CI: 2.15 – 4.30). Generally, 3.1% of the adolescents had experience using narcotics such as opium and marijuana. A total of 1.3% of the adolescents had experienced glass or crack, with 100% of them being current users. Factors associated with the experience of alcohol use for each sex, using multivariate logistic regression analysis (Backward-LR model). The results of our multivariate logistic regression indicate that low parental control rather than medium control [AOR, 95% CI, 0.09 (0.03–0.31), P < 0.001], lifetime cigarette use [AOR, 95% CI, 10.41 (3.82–28.38), P < 0.001], having a tobacco user among the friend [AOR, 95% CI, 4.36 (1.02–18.30), P < 0.001], and having an alcohol user among the friends [AOR, 95% CI, 5.84 (2.04–16.72), P < 0.01] are the factors that are significantly related to the experience of alcohol use in female adolescents (Table 2). The results of our multivariate logistic regression indicated that studying in private schools rather than public schools [AOR, 95% CI, 3.46 (1.65–7.24), P < 0.01], lifetime cigarette use [AOR, 95% CI, 3.41 (1.62–7.19), P < 0.01], lifetime water pipe use [AOR, 95% CI, 4.43 (1.69–11.61), P < 0.01], experience of sexual activity [AOR, 95% CI, 8.52 (3.88–18.74), P < 0.001], having an alcohol user among the friends [AOR, 95% CI, 12.60 (4.31–36.85), P < 0.001], and having a water pipe user in family member [AOR, 95% CI, 2.98 (1.43–6.21), P < 0.01] are the factors that are significantly related to the experience of alcohol use in male adolescents (Table 3).

**Table 1.** Alcohol and Other Drug Use Among the Adolescents, by Gender (n = 1,201)

Items	Sex				Total		OR, 95% CI
	Female		Male				
	%	CI	%	CI	%	CI	
**Lifetime alcohol use ^a, b^**	8.4	6.35–10.95	21.9	18.62–25.46	15.1	13.10–17.24	3.04 (2.15–4.30)
**Current alcohol use ^c^**	46.8	32.11–61.92	54.4	42.25–63.33	52.3	44.58–59.98	1.36 (0.69–2.66)
**Binge drinking ^d, b^**	58.3	36.64–77.89	34.8	22.79–46.31	41.1	30.84–51.98	2.62 (1.01–6.81)
**Lifetime opium and marijuana use**	2.1	1.15–3.64	4.1	2.62–5.99	3.1	2.18–4.23	0.32 (0.06–1.85)
**Current opium and marijuana use ^e^**	70	34.75–93.32	37.5	18.80–59.40	47.1	29.77–64.87	0.26 (0.05–1.26)
**Lifetime glass and crack use**	1	0.37–2.17	1.6	0.71–2.93	1.3	0.71–2	1.56 (0.55)
**Lifetime heroin use**	1.5	0.69–2.86	1.7	0.83–3.16	1.6	0.98–2.5	1.14 (0.46–2.84)
**Lifetime methamphetamines use**	2.2	1.17–3.70	3.3	1.99–5.10	2.7	1.87–3.82	1.53 (0.75–3.13)
**Lifetime ecstasy use**	5	3.41–7.10	6.2	4.38–8.45	5.6	4.36–7.07	1.25 (0.76–2.06)
**Lifetime steroid tablets use for bodybuilding**	4	2.60–5.93	6	4.25–8.31	5	3.84–6.42	1.53 (0.90–2.61)
**Lifetime Ritalin abuse**	3.5	2.19–5.32	3.3	1.99–5.06	3.4	2.43–4.59	0.93 (0.49–1.75)
**Lifetime injected illegal drugs**	2.9	1.17–4.64	3.3	2.02–5.17	3.1	2.20–4.31	1.15 (0.59–2.23)
**Receiving suggestions for narcotic use from others^b^**	13.4	10.79–16.42	18.5	15.45–21.96	15.9	13.89–18.16	1.47 (1.07–2.01)

^a^Drinking alcohol ≥ 1 day throughout the entire life

^b^p < 0.05

^c^Drinking alcohol ≥ 1 day during the last 30 days, among the 15.1% of the adolescents who had the experience of drinking alcohol

^d^Drinking alcohol 5 times or more in ≥ 1 day during the last 30 days, among the 15.1% of the adolescents who had the experience of drinking alcohol

^e^Using opium and marijuana ≥ 1 day during the last 30 days, among the 3.1% of the adolescents who had the experience of opium and marijuana use

**Table 2.** Results of our Multivariate Logistic Regression Analysis of Lifetime Alcohol Use in Female Adolescent

Variables	AOR, 95% CI ^a^	P value
**Parental control**		
Intermediate	1 (ref.)	
High	0.46 (0.09–2.43)	
Low	0.09 (0.03–0.31)^b^	< 0.001
**Lifetime cigarette use**		
No	1 (ref.)	
Yes	10.41 (3.82–28.38)^b^	< 0.001
**Having a smoker among friends**		
No	1 (ref.)	
Yes	4.36 (1.02–18.30)^b^	< 0.001
**Having an alcohol user among friends**		
No	1 (ref.)	
Yes	5.84 (2.04–16.72)^c^	< 0.01

^a^Adjusted odds ratio, 95% confidence intervals

^b^p < 0.001

^c^p < 0.01

**Table 3.** Results of our Multivariate Logistic Regression Analysis of Lifetime Alcohol Use in Female Adolescent

Variables	AOR, 95% CI ^a^	P value
**School type**		
Public	1 (ref.)	
Private	3.46 (1.65–7.24)^b^	< 0.01
**Lifetime cigarette use**		
No	1 (ref.)	
Yes	3.41 (1.62–7.19)^b^	< 0.01
**Lifetime water pipe use**		
No	1 (ref.)	
Yes	4.43 (1.69–11.61)^b^	< 0.01
**Sexual activity experience**		
No	1 (ref.)	
Yes	8.52 (3.88–18.74)^c^	< 0.001
**Having an alcohol user friends**		
No	1 (ref.)	
Yes	12.60 (4.31–36.85)^c^	< 0.001
**Having a water pipe user family**		
No	1 (ref.)	
Yes	2.98 (1.43–6.21)^b^	< 0.01

^a^Adjusted odds ratio, 95% confidence intervals

^b^p < 0.01

^c^p < 0.001

## 5. Discussion

Our findings indicate the frequency of the experience of alcohol, traditional narcotics (e.g., opium and marijuana), and psychedelics (e.g., ecstasy) to be 15.1%, 3.1%, and 5.6%, respectively, among adolescents in Tehran. Our findings differ from those of other studies conducted in Iran. Previous studies have reported the experience of alcohol use in Iranian adolescents to be as frequent as 12.6% to 32%. (6-8). The discrepancy in different studies may be accounted for by the different geographical locations where the studies have been conducted. The findings of the present study indicate that 5.6% of adolescents have experienced ecstasy at least once in their life. One study conducted in west Tehran reported a frequency of 7.6% among adolescents; however, this study focused on only one of the four areas in Tehran (north, south, west and east) and thus could not represent the entire adolescent population of Tehran (13).

A comparison of our findings with those of previous Iranian studies demonstrates the fact that our study has found a smaller frequency for substance abuse in adolescents. For instance, one study conducted on students aged 15–18 years in Tehran reported that 6.9% of students had positive test results for metabolites of morphine and hashish (14). It appears that the discrepancy between our findings and those of previous studies reflects the difference in data collection. We used self-administered questionnaires to determine the frequency of narcotics use. Since substance abuse constitutes a felony under the Iranian penal law, there is a risk of underreporting by adolescents when using self-report methods. In comparison to other countries, the use of narcotics and psychedelics is considerably less frequent in our study (15, 16). This observation may be attributed to religious and legal restrictions, as well as the social and family values in Iran that frown upon substance abuse in Iran. Our findings indicate that with increasing age, the probability of alcohol use rises, which is consistent with the findings of previous studies. Our findings indicate that the use of alcohol and the use of illegal substances are mutually related to each other and other high-risk behaviors such as smoking cigarettes and sexual behaviors. (17-21). A review of the literature reveals the fact that alcohol affects the cognitive processes and the faculty of decision making in the drinker, thus contributing to high-risk sexual behaviors (22, 23). Our findings, in line with other studies, indicate that the experience of alcohol use is strongly related to cigarette use by friends (24). An adjusted analysis of our findings indicates that studying in private schools raises the probability of alcohol use by male adolescents almost 3.5 times compared to studying in public schools. This finding probably reflects the family structure and social status of these adolescents. Iranian families with traditional structures and middle social status tend to enroll their children in public schools. Such families are generally more sensitive about the observance of religious issues, and their objection to alcohol use is greater. Studies indicate that parental objection to alcohol use is related to more limited contact of the adolescent with alcohol-drinking friends and consequently less chances of drinking alcohol by the adolescent himself/herself (25). The findings of the present study indicate that water pipe use by family members increases the chances of experiencing alcohol by adolescents by almost 3 times. Numerous studies have indicated that substance abuse by parents increases the risk of alcohol and other drugs use in their children (26). Our findings indicate that in girls, low parental control is associated with the reduced experience of alcohol use, which is inconsistent with other studies (27, 28). Moreover, high parental control increases the risk of alcohol use in boys by almost 5 times. These discrepancies may be due to the fact that high parental control probably leads to low parental attachment in adolescents. One study indicated that adolescents with low parental attachment are 11 times more likely to drink alcohol (25). The major strength of our study is our use of a standard questionnaire. This allows us to compare our findings with those of other studies conducted worldwide. In addition, our study is population-based; therefore, those adolescents who participated in our study represent the adolescent population of Tehran. In general, certain obstacles limit our study. Cultural barriers necessitated the use of self-administered questionnaires. The sensitive nature of the issue under study raises the risk of underreporting substance abuse by adolescents.30 Therefore, we used anonymous questionnaires to circumvent this problem and prevent systematic errors. Sensitivities on the parents’ part made the access to adolescents, particularly girls, rather difficult; we provided reassurance to the parents about the confidentiality of the information to encourage their participation. Although our findings illustrate the prevalence of alcohol and substance abuse among adolescents in Tehran, it may be expected that these figures are lower than the real statistics owing to the sensitive nature of substance abuse. Our findings indicate a higher frequency for the use of psychedelics, such as ecstasy, compared to traditional narcotics, such as opium and marijuana. Furthermore, dependence on artificial narcotics was higher, with 100% of individuals who had experienced glass and crack being current users. Considering the sex discrepancies observed in the factors influencing the experience of alcohol use, we recommend interventional plans aimed at improving adolescent health regarding substance abuse to take into account gender differences and the Islamic Iranian culture. Although the findings of this study are important for the authorities in public health, it is also essential to conduct future studies on a national scale with larger sample sizes.
